# Mediating Role of Psychological Inflexibility as Transdiagnostic Factor in the Relationship Between Emotional Dysregulation and Sleep Problems With Symptoms of Emotional Disorders

**DOI:** 10.3389/fpsyg.2022.800041

**Published:** 2022-04-25

**Authors:** Farrin Orouji, Reza Abdi, Gholamreza Chalabianloo

**Affiliations:** ^1^Department of Psychology, Azarbaijan Shahid Madani University, Tabriz, Iran; ^2^Department of Psychology, Faculty of Education and Psychology, Azarbaijan Shahid Madani University, Tabriz, Iran

**Keywords:** psychological inflexibility, sleep problems, emotional dysregulation, emotional disorders, mediating role

## Abstract

This study aims to investigate the mediating role of psychological inflexibility as a transdiagnostic factor in the relationship between emotional dysregulation and sleep problems with symptoms of emotional disorders. A total of 500 subjects from three universities were selected by random multistage clustering, and they completed the Pittsburgh Sleep Quality Index, Difficulties in Emotional Regulation Scale, and Acceptance and Action Questionnaire–II, Inventory of Depression and Anxiety Symptoms. The results of correlation coefficients revealed that there is a positive and significant correlation among emotional dysregulation, sleep problems, and psychological inflexibility with emotional disorders. In addition, the results showed that psychological inflexibility acts as a transdiagnostic factor that mediates the relationship between emotional dysregulation and sleep problems with symptoms of emotional disorders. These findings illustrate how emotional dysregulation and sleep problems affect emotional disorders through psychological inflexibility.

## Introduction

Emotions play an important role in human life. All humans experience a variety of emotions in their lifetime both consciously and unconsciously (Adam, 2007). Emotions emerge because of the adaptive response of various systems in working together effectively; therefore, emotions help people respond effectively to important life challenges and opportunities, and their dysfunction may cause emotional disorders (Gross, 2008). Emotional disorders are prevalent psychological disorders associated with the severity of anxiety, depression, fear, and physical symptoms (Goldberg et al., 2009). Anxiety and depression have common biological and psychological vulnerability factors and mediating mechanisms (Chorpita and Barlow, 1998). Previous studies have reported a relationship among emotional dysregulation, sleep problems (Fairholme et al., 2013), and psychological inflexibility (Levin et al., 2014) with emotional disorders (Orouji, 2018). Significant comorbidity rates of anxiety and depression in adults (Mineka et al., 1998) and youths (Brady and Kendall, 1992; Seligman and Ollendick, 1998) have been estimated. Nowadays, there is an increasing interest in clinical psychology on the identification of transdiagnostic factors that cause a wide array of disorders (Mansell et al., 2009; Nolen-Hoeksema and Watkins, 2011). Recognizing the similarities among disorders is important in assessing comorbidities among them (Levin et al., 2014). Moreover, this comorbidity is often found among psychological disorders (Kessler et al., 2005), which suggests that these problems may have common pathological processes. Therefore, considering problems that emotional disorders cause and studying the pathology of these disorders, and identifying transdiagnostic factors are important.

Psychological flexibility is defined as open acceptance of unpleasant feelings, thoughts, and emotions (Hayes et al., 1999), and includes six underlying processes: acceptance, contact with the present moment, cognitive defusion, sense of self as observer, values-based action, and committed action (Hayes et al., 2006). Mindfulness or focusing on the present moment allows a person to act in the appropriate context in achieving their goals and values (Hayes et al., 1999). In turn, psychological inflexibility consists of dysfunctional control efforts that are named as the six core psychological inflexibility processes: experiential avoidance, inflexible attention, disrupted values, inaction or impulsivity, conceptualized self, and cognitive fusion (Hayes et al., 2006; Levin et al., 2014). In psychopathology, psychological inflexibility is considered a transdiagnostic etiological factor in the development and maintenance of psychological disorders and emotional difficulties (Hayes et al., 1996). A person, who has difficulty confronting unpleasant situations through one or more of these six processes, is prone to gradually develop psychological inflexibility, which may in turn lead to emotional disorders (Tanhan, 2019). There are considerable associations between psychological inflexibility and a wide spectrum of psychological disorders marked by the prevalence of the avoidant reaction style (Bond et al., 2011). A large number of studies back up the idea that psychological inflexibility has a mediating function. A study found that psychological inflexibility mediated the relationship between stress and psychopathology, including depression, somatization, and anxiety (Arslan et al., 2021). Another study has examined the mediating role of psychological inflexibility in the relationship between fear of negative evaluation and psychological vulnerability (Uğur et al., 2021). Levin et al. (2014) examined psychological inflexibility as a transdiagnostic process across psychological disorders.

Emotional regulation refers to the ability to recognize, understand, and accept emotions, control impulsive acts in line with personal goals, and apply strategies for modulating emotional responses (Norberg et al., 2010). Adaptive emotion regulation includes many strategies for emotional regulation and flexibility in using such strategies; lack of these abilities may lead to emotional dysregulation (Ma and Fang, 2019). Strategies for emotion regulation are categorized into two groups: effective strategies, including problem-solving, re-evaluation, and acceptance; and ineffective strategies, including rumination, emotional avoidance, and suppression (Gross, 2014), which are related to anxiety and depression disorders (Aldao and Nolen-Hoeksema, 2010; Aldao et al., 2015). In contrast, emotional dysregulation refers to inflexible strategies that interfere with personal, cognitive, and social functions (Cole et al., 1994). Inefficiency in these functions present itself in the form of problems within four groups: awareness and understanding of emotions, emotional acceptance, ability to control impulsive acting according to goals in the presence of negative affect, and accessibility to efficient emotion regulation strategies (Cole et al., 1994).

Emotional dysregulation is related to anxiety disorder (Mennin et al., 2005) and depression (Nolen-Hoeksema et al., 2008). Furthermore, evidence indicates that emotional dysregulation may cause mental health problems and predict symptoms of depression (Thompson et al., 2011) and anxiety (Mennin and Farach, 2007; Nolen-Hoeksema et al., 2008). There is evidence that supported that mentally healthy participants showed similar symptoms reported in clinical samples experiencing emotional dysregulation (Petrovic et al., 2016), which shows that symptoms of emotional dysregulation can exist among the general population. Furthermore, the research of Cobos-Sánchez et al. (2020) supports the relationship between emotional regulation and psychological flexibility; they indicated that emotional regulation could influence psychological flexibility.

Sleep is an important psychological process in human life, in which disruption leads to consequences. These consequences of sleep disturbances can cause problems in cognitive, emotional, and physical functioning (Schoenborn and Adams, 2010; Baglioni et al., 2016). Sleep problems are another factor that is correlated with psychological health problems, including stress (Linton, 2004; Jansson and Linton, 2006a), anxiety (Taylor et al., 2005; Jansson and Linton, 2006b), depression (Ohayon, 2002; Cole and Dendukuri, 2003; Riemann and Voderholzer, 2003; Taylor et al., 2003), and catastrophic worries (Talbot et al., 2010). Some studies indicate the association between sleep problems and higher levels of psychological symptom severity (Lund et al., 2010; Nyer et al., 2013; Owens et al., 2014; Peltz and Rogge, 2016; Peltz et al., 2020). Sleep problems are involved in the pathology of emotional disorder symptoms (depressive symptoms) through emotional regulation (Baglioni et al., 2010). Theoretically, two processes are involved in sleep-related cognitions with sleep problems: sleep-interfering processes and sleep-interpreting processes. Sleep-interfering processes refer to stressor events, traumas, emotional conflicts, depression, worries, and negative conditioning, and cause emotional or cognitive arousal during sleep. For sleep-interpreting processes, various personal standards, views, beliefs, and fear may have effects on how a person interprets sleep modulation, sleep problems, and the consequences of not getting enough sleep (Lundh and Broman, 2000). Studies have shown that short-time sleep may result in reduced divergent thinking, increased response perseveration on ineffective solutions, and lack of awareness (Goel et al., 2009). Difficulty in cognitive functions due to sleep problems is related to processes of psychological inflexibility (Peltz et al., 2020).

**FIGURE 1:**
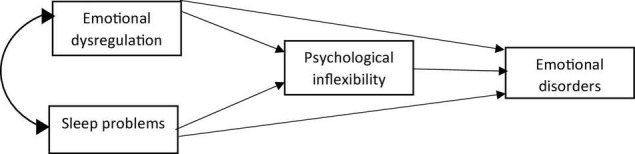
The hypothesized model of mediating role of psychological inflexibility as transdiagnostic factor in the relationship between emotional dysregulation and sleep problems with symptoms of emotional disorders.

Although some studies have addressed emotional dysregulation, sleep problems (Fairholme et al., 2013), and psychological inflexibility (Levin et al., 2014) in the prediction of severity of emotional disorder symptoms (Orouji, 2018), there are only a few studies evaluating the mediating role of psychological inflexibility. In this study, the researchers assumed that psychological inflexibility, as a transdiagnostic factor, would mediate the relationship between emotional dysregulation and sleep problems with symptoms of emotional disorders and psychological inflexibility which is considered as a mediating variable, was examined by path analysis. The results regarding the mediating effect of psychological inflexibility in the relationship between emotional dysregulation and sleep problems with symptoms of emotional disorders is shown in Figure 1.

## Method

### Participants and Procedure

This is a correlational study in which 500 students were selected with the random multistage clustering sampling method. Among the subjects, 263 (52.6%) were males and 237 (47.4%) were females. Also, 452 (90.4%) were single and 48 (9.6%) were married. First, the purpose of the study, examining the mediating role of psychological inflexibility as a transdiagnostic factor in the relationship between emotional dysregulation and sleep problems with symptoms of emotional disorders, was explained to the subjects. They were assured that their information would remain confidential.

### Measures

#### Pittsburgh Sleep Quality Index

Pittsburgh Sleep Quality Index (PSQI) was used to assess the sleep quality of the participants. Buysse et al. (1989) developed it at the Psychiatric College of Pittsburgh. It includes 19 questions devoted to investigating 7 subscales: subjective sleep quality, sleep latency, sleep duration, habitual sleep efficiency, sleep disturbances, use of sleep medication, and daytime dysfunction over the last month rated with a 4-point Likert scale (0–3). It takes 5–10 min to complete. Internal consistency is 0.83 by Cronbach’s alpha, and its reliability is 0.85. Internal validity and reliability are 0.86 and 0.89, respectively (Shahrifar, 2009). In this study, the Cronbach’s alpha coefficient of this questionnaire was higher than 0.85.

#### Difficulties in Emotional Regulation Scale

This questionnaire was developed by Gratz and Roemer (2004) to assess difficulties in emotional regulation. Its subscales include non-acceptance of emotional responses, difficulty engaging in goal-directed behavior, impulse control difficulties, lack of emotional awareness, limited access to emotional regulation strategies, and lack of emotional clarity. This scale has 36 items rated by 5 Likert points (1–5). Internal consistency is 0.93. All the six subscales have 0.8 for Cronbach’s alpha (Gratz and Roemer, 2004). Azizi et al. (2010) reported 0.92 for Cronbach’s alpha. This research applied this for assessing emotional dysregulation with a Cronbach’s alpha coefficient of higher than 0.85.

#### Acceptance and Action Questionnaire – II

This questionnaire was developed by Bond et al. (2011) for measuring psychological inflexibility and diversity of acceptance and experiential avoidance and has 7 items. The score of each person is 7. All the items were rated by 7 Likert points. A higher score represents low psychological flexibility and high experiential avoidance. The reliability of test-retest and Cronbach’s alpha are reported 0.81, 0.79, and 0.84, respectively. In Iran, Abbasi et al. (2012) reported convergence validity and accepted internal consistency. An acceptance and action questionnaire was applied to measure psychological inflexibility, with a Cronbach’s alpha coefficient of 0.85.

#### Inventory of Depression and Anxiety Symptoms

This questionnaire was designed by Watson et al. (2007) and has 64 items, which evaluate 10 factors related to anxiety and depression. The factors include suicidality, lassitude, insomnia, appetite loss, appetite gain, ill-temper, well-being, panic, social anxiety, and traumatic intrusions. This questionnaire is scored based on Likert rating scales (from 1 to 5; Jorbonian, 2015). This questionnaire was completed by 303 students as well as 605 mental patients. The means of convergence correlation were calculated as 0.51 and 0.62, respectively. All the subscales except for welfare had high validity (Watson et al., 2007). This questionnaire was standardized and implemented by Jorbonian (2015) in Iran where the formal and content validities were evaluated by two faculty members of Azerbaijan Shahid Madani University. Reliability was calculated with a Cronbach of 0.95. This research applied this questionnaire for depression and anxiety with a Cronbach’s alpha coefficient of 0.85.

### Data Analysis

To test the research model, a path analysis was conducted using the Amos statistical software. Before the path analysis, single-variable and multivariable missing data were excluded. The skewness and kurtosis of data distribution were calculated with the results, showing that none of them were higher than ± 1. The results suggested normality of data distribution (*p* > 0.05). Independence of errors for regression equations was examined by the Durbin Watson test, with the calculated number approving this assumption. Collinearity was not observed. In addition, variance and tolerance inflation factors were calculated for multi-collinearity and given two factors, but no multi-collinearity was revealed.

## Results

### Descriptive Statistics

In this section, descriptive findings are provided first. Then, results of the path analysis are presented to examine the hypothesis. Descriptive data include standard deviation of variables as well as correlation coefficients (Table 1).

**TABLE 1:** Correlation matrix, means, and standard deviation of research variables.

	Mean	SD	1	2	3	4
1-Emotional dysregulation	104.24	20.479	1			
2-Sleep problems	6.92	1.384	0.477**	1		
3-Psychological inflexibility	21.24	3.871	0.597**	0.504**	1	
4-Emotional disorders	144.43	18.429	0.541**	0.459**	0.575**	1

significant at 0.01 level**.

Table 1 shows the means and standard deviations, and the correlations among emotional dysregulation, sleep problems, psychological inflexibility, and emotional disorders. According to these results, emotional dysregulation, sleep problems, and psychological inflexibility are significantly positively correlated with emotional disorders (*p* < 0.01).

### Mediating Role of Psychological Inflexibility

**FIGURE 2:**
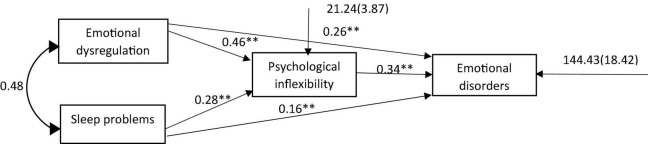
Standardized path coefficients of the hypothesized model. **P < 0.01.

In Figure 2, standardized path coefficients are shown to evaluate the mediating role of psychological inflexibility in the relationship between emotional dysregulation and sleep problems with emotional disorders. To test the potential bidirectional relationship between the predictive and mediating variables, the direction of the relationship is reversed and examined in two competitive models.

**FIGURE 3:**
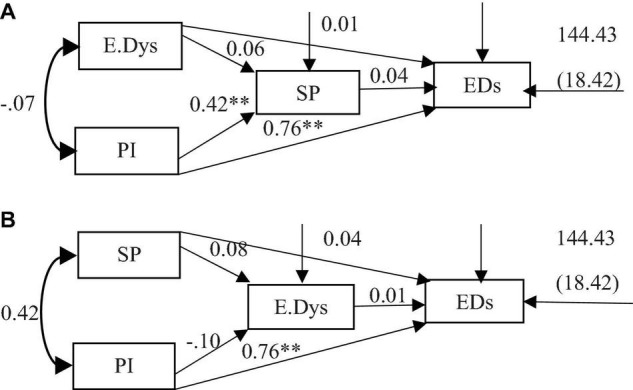
**(A,B)** The competitive models. **P < 0.01. E.Dys, emotional dysregulation; PI, psychological inflexibility; SP, sleep problems; Eds, emotional disorders.

As shown in Figure 3A, root mean square error approximation (RMSEA) was equal to 1.117, and IFI, GFI, CFI, and NFI were much smaller than those in the criteria (0.9). The obtained coefficients indicate that the model does not indicate good fit. As shown in Figure 3B, RMSEA was equal to 1.162, and IFI, GFI, CFI, and NFI were much smaller than those indicated in the criteria (0.9). The obtained coefficients do not indicate good model fit.

Table 2 presents the indices of model fit. RMSEA was equal to 0.64, and second root of the mean of remaining squares (SRMR) was equal to 0.051, which is lower than that in the criteria (0.08); thus, the fitness of the model is approved. IFI, GFI, CFI, and NFI were higher than those in the criteria (0.9), which indicated good model fit.

**TABLE 2:** Model fit indices.

Fit index	Accepted range	Observed value	Evaluation of fit index
IFI	>0.9	0.947	Good
GFI	>0.9	0.941	Good
RMSEA	<0.08	0.064	Good
SRMR	<0.08	0.051	Good
CFI	>0.9	0.946	Good
NFI	>0.9	0.942	Good

The relationships between variables are shown in Table 3.

**TABLE 3:** Direct effects, indirect effects, and total effects in the final model.

From variable	To variable	Direct effects	Indirect effects	Total effects	Explained variance
Emotional dysregulation	Psychological inflexibility	0.461**	_	0.461**	0.419
Sleep problems		0.285**	_	0.285**	
Emotional dysregulation		0.262**	0.155**	0.417**	
Sleep problems	Emotional disorders	0.165**	0.095**	0.260**	0.410
Psychological inflexibility		0.335**	_	0.335**	

significant at level 0.01**.

The assessment in Table 3 shows that emotional dysregulation has a significant direct effect on psychological inflexibility (*p* < 0.01, β = 0.461), and that sleep problems has a significant direct effect on psychological inflexibility (*p* < 0.01, β = 0.285).

Emotional dysregulation showed direct and positive effects (*p* < 0.01, β = 0.262) as well as an indirect effect (*p* < 0.01, β = 0.155) on emotional disorders through psychological inflexibility. Sleep problems showed direct and positive effects (*p* < 0.01, β = 0.165) as well as an indirect effect (*p* < 0.01, β = 0.095) on emotional disorders through psychological inflexibility. Psychological inflexibility revealed a direct effect on emotional disorders (*p* < 0.01, β = 0.335).

## Discussion

According to the findings of this study, psychological inflexibility, as a transdiagnostic factor, has a mediating role in the relationship between emotional dysregulation and sleep problems with symptoms of emotional disorders. The findings showed that psychological inflexibility is related to symptoms of emotional disorders, which is consistent with other previous studies demonstrating the transdiagnostic role of psychological inflexibility in various types of psychological problems (Hayes et al., 2006; Kashdan et al., 2009; Giorgio et al., 2010; Newman and Llera, 2011; Fledderus et al., 2012; Levin et al., 2014; Uğur et al., 2021).

Psychological inflexibility plays an important role in the development, maintenance, and exacerbation of psychological problems. Given the fact that the type of problematic behaviors emerges differently in various disorders, many of them can be conceptualized as common psychological concepts (Hayes et al., 1996). Problematic behaviors that are the cause of many psychological disorders have common avoidance functions; these disorders have a similar pattern of inflexible reaction instead of values-based action in confronting negative feelings and thoughts (Levin et al., 2014). Therefore, it seems that people with this problem fail to achieve their goals and values because of the dominance of inflexible psychological reactions over flexible reactions to a present situation, and replication of this cycle may increase their vulnerability to anxiety and depression. Also the relationship between experiential avoidance or rejection of emotional responses and negative emotions (Gratz and Roemer, 2004) predicts vulnerability to anxiety and depression where people who have a high level of negative effectivity may be anxious and worried to exposure with negative emotions (Thomas et al., 2013), and their abilities for evaluating situation and selecting efficient strategies will be lost (Pollock et al., 2016). When a person uses experiential avoidance, avoids private thoughts, feelings, and emotions, and is unwilling to stay in contact with inner and private thoughts, feelings, and emotions (pleasant or unpleasant), this may lead to suffering (Hayes et al., 2006; Tanhan, 2019). Subsequently, when there is a tendency to escape or avoid inner experiences, it is likely that unwanted experiences will turn into psychological problems through psychological inflexibility over time (Greco et al., 2008; Hayes et al., 2012).

The findings of this research suggested that emotional dysregulation is related to symptoms of emotional disorders directly, which is consistent with previous studies (Levitt et al., 2004; Mennin et al., 2005; Turk et al., 2005). Emotional dysregulation occurs when control of negative emotions in stressful circumstances is not sufficiently counterbalanced by positive and pleasurable feelings, resulting in incapacity to endure powerful, unpleasant, and prolonged emotional states, according to many research (Van Beveren et al., 2019; Waugh, 2020). Moreover, persistence of this state can easily develop symptoms of depression and anxiety (Van Beveren et al., 2019). However, when this cycle is triggered, the use of maladaptive strategies will increase, and psychological health will be affected (Torres et al., 2013; Koob, 2015).

Also, the findings of this study revealed that emotional dysregulation has an indirect relationship with symptoms of emotional disorders by mediating the role of psychological inflexibility. Recent findings showed a positive relationship between emotional dysregulation and psychological inflexibility (Cobos-Sánchez et al., 2020). People who have difficulty regulating emotions seem to be unable to control impulses in certain emotional situations, are unaware of their emotions, have difficulty achieving goals in distressing situations, do not accept negative emotions, use limited emotion regulation strategies, and may experience a long period of distress (Mennin and Farach, 2007; Nolen-Hoeksema et al., 2008). As a result, they may use the skills of psychological inflexibility in these situations; they either try to avoid distressing situations or they attach a thought to an experience and refuse to take positive actions, since they have not achieved the desired result in similar situations before (Cobos-Sánchez et al., 2020). In other words, the person who has difficulty managing and regulating emotions, in addition to these issues, their conduct demonstrates psychological inflexibility (Cobos-Sánchez et al., 2020) which leads to increasing symptoms of various forms of psychopathology mainly anxiety and depression (Aldao et al., 2010; Kashdan and Rottenberg, 2010). Therefore it is thought that emotional dysregulation has an indirect effect on emotional disorders through psychological inflexibility due to the fact that emotional dysregulation and psychological inflexibility are factors related to psychopathology (Aldao et al., 2016).

Also, these findings showed direct and significant relationships between sleep problems and symptoms of emotional disorders. These findings are consistent with previous studies (Benca et al., 1992; Taylor et al., 2005; Harvey et al., 2011; Peltz et al., 2020). Sleep problems increase one’s emotional reactivity to intense and persistent emotional arousal by disrupting emotional regulation processes (Baglioni et al., 2010), which will increase one’s vulnerability to psychological disorders and development of emotional disorder symptoms (Baglioni et al., 2016). Also, the indirect effect of sleep problems on symptoms of emotional disorders through psychological inflexibility was examined; it is said that poor sleep affects self-regulatory behaviors (Baglioni et al., 2010), which means it might impair the capacity to regulate emotions and increase emotional reactivity (Ohayon, 2002; Baglioni et al., 2010; Tavernier and Willoughby, 2014). Therefore, poor sleep might be associated with using flexible and inflexible skills when handling uncomfortable or unwanted thoughts, feelings, and experiences. Moreover, poor sleep might promote the use of psychologically inflexible skills and reduce or interfere with the use of psychologically flexible skills, thereby increasing the potential for psychological distress, such as depressive symptoms (Peltz et al., 2020).

Also, the findings of our study are consistent with research studies that indicated sleep problems may disrupt cognitive flexibility, may reduce the capacity of psychological flexibility, and may increase psychological inflexibility (Couyoumdjian et al., 2010), which will eventually increase the symptoms of emotional disorders (Peltz et al., 2020).

This study has some limitations. The first is that the subjects selected were normative college students; therefore, generalization of the research findings to other population (such as clinical population) should be conducted with caution. The second is the possibility that there are some other mediators originating from more contextual conditions. Third, the lack of an appropriate validated measurement scale for the Iranian population to determine the severity of sleep problems requires more research and instrument development or adaptation. Additionally, due to the fact that the present study is a cross-sectional design to explore the possible relationship but not causal inference. We cannot determine the causal role of psychological inflexibility, and prospective studies will help to further establish the nature of the relationships we observed here. Experimental studies may also, in the future, indicate a bi-directional relationship between psychological flexibility as a complex transdiagnostic psychological construct and other variables.

In conclusion, this study found that psychological inflexibility mediates the relationship between emotional dysregulation and sleep problems with symptoms of emotional disorders. Both emotional dysregulation and sleep problems had a significant impact on psychological inflexibility, and increases in dimensions of psychological inflexibility were associated with increases in emotional disorder symptoms. The findings contribute to the related literature in research.

## Data Availability Statement

The original contributions presented in the study are included in the article/supplementary material, further inquiries can be directed to FO, fn.oi0720@gmail.com

## Ethics Statement

Ethical review and approval was not required for the study on human participants in accordance with the local legislation and institutional requirements. Written informed consent for participation was not required for this study in accordance with the national legislation and the institutional requirements.

## Author Contributions

RA contributed to the design of the study. FO performed the data collection and analysis and wrote the manuscript. All authors read and approved the final version of the manuscript.

## Conflict of Interest

The authors declare that the research was conducted in the absence of any commercial or financial relationships that could be construed as a potential conflict of interest.

## Publisher’s Note

All claims expressed in this article are solely those of the authors and do not necessarily represent those of their affiliated organizations, or those of the publisher, the editors and the reviewers. Any product that may be evaluated in this article, or claim that may be made by its manufacturer, is not guaranteed or endorsed by the publisher.
